# Community‐Acquired Pneumonia in Young Children: Variables Associated With Emergency Department Attendance and Hospitalization

**DOI:** 10.1155/ijpe/8884147

**Published:** 2026-06-30

**Authors:** Shay Nemet, Yael Reichenberg, Avner Herman Cohen, Shai Ashkenazi, Bernice Oberman, Yoel Levinsky, Moriya Cohen, Eli Cohen, Vered Shkalim Zemer

**Affiliations:** ^1^ Allergy and Clinical Immunology Unit, Kaplan Medical Center, Rechovot, Israel, kmc.org.il; ^2^ Pediatric Department, Faculty of Medicine, Hebrew University of Jerusalem, Jerusalem, Israel, huji.ac.il; ^3^ Pediatric Department, Clalit Health Services, Dan-Petach Tikva District, Israel, clalit.co.il; ^4^ Pediatric Department, Faculty of Medical & Health Sciences, Tel Aviv University, Tel Aviv, Israel, tau.ac.il; ^5^ Pediatric Department, Pediatric Ambulatory Community Clinic, Petach Tikva, Israel; ^6^ Pediatric Department, Adelson School of Medicine, Ariel University, Ariel, Israel, ariel.ac.il; ^7^ Pediatric Rheumatology Unit, Schneider Children′s Medical Center, Petach Tikva, Israel; ^8^ Department of Pediatrics B, Schneider Children′s Medical Center, Petach Tikva, Israel; ^9^ Microbiology Unit, Ariel University, Ariel, Israel, ariel.ac.il; ^10^ Microbiology Unit, Bar Ilan University, Israel, biu.ac.il

**Keywords:** antibiotic stewardship, children, community-acquired pneumonia, guidelines, hospitalization

## Abstract

**Aim:**

This study was aimed at identifying variables associated with attending pediatric emergency departments (PEDs) and hospitalization of young children with community‐acquired pneumonia (CAP).

**Methods:**

A population‐based case‐control study, which included all children aged 1–6 years diagnosed with CAP from three major districts of a health maintenance organization during 2014–2020. Children born preterm, or with chronic lung diseases or immunodeficiency, were excluded. The cohort was divided into three groups: children diagnosed and treated by the community pediatrician, those who attended PEDs, and those hospitalized.

**Results:**

The cohort comprised 59,662 children with CAP, of whom 3685 (6.2%) attended PEDs and 2026 (3.4%) were hospitalized. Of the children treated in the PED, 33% were hospitalized versus 1.4% of those diagnosed in the community. Using multivariable analyses, members of a minority sector, low or middle socioeconomic status, elevated white blood cell count, and high C‐reactive protein levels were independently and significantly associated with PED attendance and hospitalization.

**Conclusion:**

Community pediatricians should integrate the identified variables associated with attending PEDs and hospitalization to optimize management of children with CAP, given the potential for complications and mortality. The recognized 6.2% PED attendance and 3.4% hospitalization, with the identified related associations, have implications for pediatric healthcare planning.

## 1. Introduction

Community‐acquired pneumonia (CAP) is one of the most common causes of morbidity and mortality in children aged ≤ 5 years, with an annual 700,000 deaths reported worldwide. It is a prevalent cause for attending primary care physicians, both pediatricians and family physicians, with associated antibiotic utilization and hospitalizations [1–3]. It is estimated that CAP accounts for 24.8% of all hospitalizations and 15.5% of all deaths in the pediatric population [4, 5].

Clinical diagnosis of CAP can be challenging, as symptoms and signs vary with age and may be nonspecific in young children [6]. Respiratory viruses are common causes of CAP in children, accounting for 30%–67% of hospitalized cases. Respiratory syncytial virus is the leading viral pathogen, detected in 30% of viral CAP. Other viral pathogens are the influenza virus, rhinovirus, parainfluenza virus, human metapneumovirus, bocavirus, adenovirus, and coronaviruses. *Streptococcus pneumonia* is the most prevalent bacterial cause across all ages, accounting for 30%–40% of cases [7–9]. Other bacterial pathogens include *Staphylococcus aureus* and *Haemophilus influenzae* type b. *Mycoplasma pneumoniae* is a common cause of atypical CAP, which accounts for 8%–40% of all CAP cases [8].

The Pediatric Infectious Diseases Society and Infectious Diseases Society of America (PIDS/IDSA) published in 2011 guidelines for the management of children with suspected CAP [10], as did the British Thoracic Society and the Israeli Pediatric Association in 2011 and 2013, respectively [11, 12]. These guidelines uphold that clinical, laboratory, and radiological criteria are unreliable tools for distinguishing between bacterial and viral etiology in pediatric CAP [10–12]. However, in the real world, there is widespread variation in common management approaches for CAP in the pediatric population in both hospital‐based and community settings [13–15]. Our study aimed to characterize CAP in a large cohort of young children and to identify real‐life variables associated with attendance of pediatric emergency departments (PEDs) and hospitalization of young children with CAP in order to augment our knowledge, incorporate these variables into clinical judgment, and hence improve the management of these very common childhood diseases.

## 2. Methods

### 2.1. Study Design

A large population‐based case‐control study was conducted among young children with CAP. The inclusion criteria were children aged 1–6 years, a diagnosis of CAP during the period of January 1, 2014, to December 31, 2020, members of the Clalit health maintenance organization (HMO), and residence in one of three major administrative Clalit HMO districts in central Israel. Exclusion criteria were children who were born preterm (< 37 weeks′ gestational age), children with chronic lung diseases, and those with underlying immune deficiency. The study cohort was divided into three groups: children who attended their pediatrician at the community clinics, those who attended a PED, and those who were hospitalized. Multiple sociodemographic, clinical, and laboratory variables were compared between the groups to elucidate variables associated with attending PEDs and hospitalization.

Clalit HMO is the largest HMO in Israel, insuring ~5 million individuals, namely, ~54% of the Israeli population. The three districts included in the present study comprised ~1.5 million Clalit HMO members, most of whom live in urban areas. Clalit HMO serves as both an insurer and a healthcare provider, operating through community clinics and hospitals. In community children′s centers, it is possible to take blood specimens and administer intravenous (IV) antibiotics under observation. Clalit HMO comprises a comprehensive computerized dataset, including subjects′ demographics, community and outpatient visits, laboratory test results, hospitalizations, medication prescriptions, and purchases. During each physician visit, a diagnosis is defined according to the International Classification of Diseases, Ninth Revision (ICD‐9). We used the MDClone analytics platform for data collection from Clalit HMO electronic medical records. The study was conducted following the Declaration of Helsinki and was approved by the Institutional Review Board of Clalit HMO (Approval Number 0040‐20‐COM).

### 2.2. Data Collection

Data retrieved from the electronic database included demographics (age, sex, sector, and socioeconomic status [SES]), auxiliary tests (complete blood count and C‐reactive protein [CRP]), antibiotic treatment, visits to PEDs, and hospitalizations. SES was defined according to the Israeli Central Bureau of Statistics classification system, which assigns 10 categories of SES based on diverse variables, such as demographic characteristics, educational attainment, employment, income levels, and housing conditions [16].

### 2.3. Statistical Analysis

Our study data were extracted into a central database and anonymized for statistical analyses. We first used descriptive statistics to report the demographic and clinical variables of the study individuals. The continuous variables were represented by mean, median, standard deviation (SD), and interquartile range (IQR) and compared by *t*‐test. The categorical variables were represented in proportions and compared using the chi‐square test. Multivariate regression analyses were then used to identify independent variables associated with PED attendance and hospitalization. A *p* value of < 0.05 was considered statistically significant.

## 3. Results

### 3.1. Characteristics and Outcome of the Study Group

A total of 59,662 children aged 1–6 years were diagnosed with CAP during the study period, with a mean age of 2.6 years. CAP was significantly more prevalent among children aged 1–2 years, who comprised 68.2% (40,672/59,662) of the cohort, compared to 24.4% (14,565/59,662) and 7.4% (4,425/59,662) of children aged 3–4 and 5–6 years, respectively (*p* < 0.001). Males were more frequently represented than females, both in those diagnosed in the community (53.4%) and in those diagnosed in the PEDs (55%), but the difference did not reach statistical significance (*p* = 0.056). The majority of patients in both groups were of the non‐ultra‐Orthodox Jewish sector (55,674, 93.3%) and of the high SES (27,952, 46.9%).

**Figure 1:**
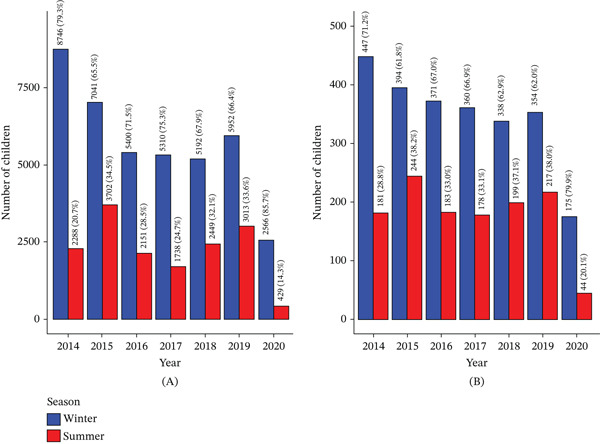
Seasonality of children diagnosed with community‐acquired pneumonia (A) in the community and (B) in emergency departments during the study period.

Of the entire study cohort, 55,977 (93.8%) were diagnosed by their pediatrician at community clinics, and 3685 (6.2%) attended a PED. Of all the children included in the study, 2026 (3.4%) were hospitalized; however, only 1.4% of the children diagnosed by the pediatricians were hospitalized compared to 33.6% of children attending the PEDs. There was seasonal variability in CAP diagnosis throughout the study: The majority of children diagnosed in both the community and PEDs were diagnosed during winter, and a minority were diagnosed during summer, as presented in Figure 1. There were some differences in the number of CAP cases over the study period, with a very low number of cases during the winter of 2020, a well‐known consequence of the COVID‐19 pandemic (Figure 1).

### 3.2. Variables Associated With Attending PEDs

Table 1 demonstrates the sociodemographic variables of the study cohort with respect to the site attendance of the children with CAP, namely, the community or a PED. While 6.2% of the young children attended PEDs, variables with a significantly (*p* < 0.001) higher PED attendance rate included children aged 1–2 years (6.7% attendance), ultra‐Orthodox Jews (8.2%) or Arab (11.1%) sectors, and low SES (8.6%). Rates of attendance also varied by the year of diagnosis. Numerical details are shown in Table 1.

**Table 1:** Sociodemographic characteristics of young children with community‐acquired pneumonia (*N* = 59,662) by site of attendance.

Variable	Category	Community pediatrician (55,977) (93.8%)	Pediatric emergency department (3685) (6.2%)	*p* value
Sex, *N* (%)	Female	26,100 (94.0%)	1658 (6.0%)	0.056
	Male	29,877 (93.6%)	2027 (6.4%)	
Age, mean (SD) (year)		2.61 (1.3)	2.35 (1.3)	< 0.001
Sector, *N* (%)	Non‐ultra‐Orthodox Jews	52,395 (94.1%)	3279 (5.9%)	< 0.001
	Ultra‐Orthodox Jews	1157 (91.8%)	104 (8.2%)	
	Arabs	2425 (88.9%)	302 (11.1%)	
Socioeconomic status, *N* (%)	High	26,326 (94.2%)	1626 (5.8%)	< 0.001
	Medium	18,537 (94.7%)	1038 (5.3%)	
	Low	9823 (91.4%)	928 (8.6%)	
Year of study, *N* (%)	2014	11,034 (94.6%)	628 (5.4%)	0.001
	2015	10,743 (94.4%)	638 (5.6%)	
	2016	7551 (93.2%)	554 (6.8%)	
	2017	7048 (92.9%)	538 (7.1%)	
	2018	7641 (93.4%)	537 (6.6%)	
	2019	8965 (94.0%)	571 (6.0%)	
	2020	2995 (93.2%)	219 (6.8%)	

*Note:*Values are presented as *N* (percentage), unless otherwise stated.

Table 2 shows the laboratory results and performance of chest radiographs in the children with CAP by site of attendance. The white blood cell count was significantly higher (*p* < 0.001) in children who had attended PEDs than in those attending the community pediatrician, both in terms of means (17,120/*μ*L vs. 12,680/*μ*L) and medians (16,230/*μ*L vs. 11,400/*μ*L). Likewise, serum CRP levels were significantly higher in children attending PEDs (mean of 8.54 vs. 5.98 mg/dL, *p* < 0.001). Chest radiographs were performed in 91.3% of the children attending PEDs versus only 23.4% of those diagnosed with CAP and treated by the community pediatricians (*p* < 0.001). Additional numerical data are presented in Table 2.

**Table 2:** Laboratory results and performance of chest radiographs in children with community‐acquired pneumonia (*N* = 59,662) by site of attendance.

Variable^a^	Community pediatrician (55,977) (93.8%)	Pediatric emergency department (3685) (6.2%)	*p* value
WBC (×10^3^/*μ*L), mean (SD)	12.68 (5.89)	17.12 (7.57)	< 0.001
WBC (×10^3^/*μ*L), median [IQR]	11.40 [8.30, 16.00]	16.23 [11.42, 21.86]	< 0.001
Serum CRP (mg/dL), mean (SD)	5.98 (5.28)	8.54 (7.04)	< 0.001
Serum CRP (mg/dL), median [IQR]	4.2 [2.3, 7.9]	6.3 [3.0, 11.8]	< 0.001
Performance of chest radiographs	13,107 (23.4%)	3364 (91.3%)	< 0.001

Abbreviations: CRP, C‐reactive protein (normal range: 0–0.5 mg/dL); WBC, white blood cell count.

^a^Values are presented as *N* (percentage), unless otherwise specified.

### 3.3. Variables Associated With Hospitalization

Only 3.4% (2026/59,662) of the children with CAP were hospitalized. Table 3 presents the sociodemographic variables of the study cohort by hospitalization or no hospitalization. Variables associated with a highly significant (*p* < 0.001) hospitalization rate included age of 1–2 years (3.7% hospitalization), ultra‐Orthodox Jews (3.6%), and Arab (7.9%) sectors. Children with CAP of the low SES had a high hospitalization rate of 5.9% compared to 2.6% in children with a high SES (*p* < 0.001). Rates of hospitalizations also varied mildly by the year of diagnosis. Detailed numerical data are presented in Table 3.

**Table 3:** Sociodemographic variables associated with hospitalization of young children with community‐acquired pneumonia.

Variable	Category	Total (59,662) (100%)	Not hospitalized (57,636) (96.6%)	Hospitalized (2026) (3.4%)	*p* value
Sex, *N* (%)	Female	27,758	26,851 (96.7%)	907 (3.3%)	0.112
	Male	31,904	30,785 (96.5%)	1119 (3.5%)	
Age, mean (SD) (years)		2.59 (1.30)	2.60 (1.30)	2.32 (1.29)	< 0.001
Sector, *N* (%)	Non‐ultra‐Orthodox Jews	55,674	53,910 (96.8%)	1764 (3.2%)	< 0.001
	Ultra‐Orthodox Jews	1261	1215 (96.4%)	46 (3.6%)	
	Arabs	2727	2511 (92.1%)	216 (7.9%)	
Socioeconomic status, *N* (%)	High	27,952	27,222 (97.4%)	730 (2.6%)	< 0.001
	Medium	19,575	18,980 (97.0%)	595 (3.3%)	
	Low	10,751	10,114 (94.1%)	637 (5.9%)	
Year of study, *N* (%)	2014	11,662	11,259 (96.5%)	403 (3.5%)	< 0.001
	2015	11,381	11,068 (87.2%)	313 (2.8%)	
	2016	8105	7838 (96.7%)	267 (3.3%)	
	2017	7586	7301 (96.2%)	285 (3.8%)	
	2018	8178	7863 (96.1%)	315 (3.9%)	
	2019	9536	9207 (96.5%)	329 (3.5%)	
	2020	3214	3100 (96.5%)	114 (3.5%)	
Site of diagnosis, *N* (%)	Community pediatrician	55,977	55,189 (98.6%)	788 (1.4%)	< 0.001
	Pediatric emergency department	3685	2447 (66.4%)	1238 (33.6%)	

The performance of blood tests, their results, and the performance of chest radiographs in the children with CAP, with hospitalization or without hospitalization, are shown in Table 4. Auxiliary tests were performed significantly (*p* < 0.001) more commonly in hospitalized children compared to those not hospitalized, including complete blood count (73.7% vs. 12.0%), serum CRP (63.8% vs. 6.0%), and chest radiographs (71.6% vs. 26.1%). The white blood cell count was significantly higher in hospitalized children than in those treated on an ambulatory basis, with means of 16,560/*μ*L versus 13,790/*μ*L (*p* < 0.001), as were the means of absolute neutrophil counts 11,0930/*μ*L versus 7520/*μ*L (*p* < 0.001). Likewise, serum CRP levels were significantly higher in hospitalized children (mean of 9.0 vs. 6.89 mg/dL, *p* < 0.001). Additional numerical data are presented in Table 4.

**Table 4:** Laboratory and imaging variables associated with hospitalization of young children with community‐acquired pneumonia.

Variable	Total (59,662) (100%)	Not hospitalized (57,636) (96.6%)	Hospitalized (2026) (3.4%)	*p* value
Performance of WBC, *N* (%)	8438 (14.1%)	6945 (12.0%)	1493 (73.7%)	< 0.001
Performance of CRP, *N* (%)	4753 (8.0%)	3461 (6.0%)	1292 (63.8%)	< 0.001
Performance of chest radiograph, *N* (%)	16,471 (27.6%)	15,020 (26.1%)	1451 (71.6%)	< 0.001
WBC (×10^3^/*μ*L), mean (SD)	14.28 (6.88)	13.79 (6.54)	16.56 (7.89)	< 0.001
CRP, mean (SD) (mg/dL)	7.42 (6.46)	6.89 (5.83)	9.00 (7.84)	< 0.001
ANC (×10^3^/*μ*L), mean (SD)	8.19 (5.51)	7.52 (4.95)	11.09 (6.71)	< 0.001
WBC (×10^3^/*μ*L), median [IQR]	12.95 [9.00, 18.20]	12.40 [8.80, 17.62]	15.19 [10.56, 21.55]	< 0.001
CRP, median [IQR] (mg/dL)	5.20 [2.65, 9.87]	5.02 [2.62, 9.07]	5.88 [2.77, 13.52]	< 0.001
ANC (×10^3^/*μ*L), median [IQR]	6.80 [4.10, 10.80]	6.30 [3.90, 10.00]	9.80 [5.80, 14.85]	< 0.001

Abbreviations: ANC, absolute neutrophil count; CRP, C‐reactive protein (normal range: 0–0.5 mg/dL); WBC, white blood cell count.

### 3.4. Multivariate Analyses

The variables identified by the descriptive univariate analyses (Tables 1, 2, 3, and 4) were included in the multivariate model to identify independent associations of PED attendance and hospitalization while controlling for confounders. The numerical results of the multivariate analyses are demonstrated in Table 5. We found that minority sectors, low and middle SES, elevated white blood cell count, high CRP levels, and performance of chest radiographs were independently associated with attending PEDs and hospitalization. Age, sex, and year of study were not independent significant associations in the multivariate models (Table 5).

**Table 5:** Multivariate analyses of variables associated with attending a pediatric emergency department (PED) or hospitalization of children with community‐acquired pneumonia (*N* = 59,662).

	Attending PED			Hospitalization		
Variable	Adjusted OR	95% CI	*p*value	Adjusted OR	95% CI	*p* value
Male sex	0.966	0.829–1.126	0.659	0.984	0.833–1.163	0.854
Age	0.949	0.894–1.008	0.087	0.985	0.896–1.024	0.215
Sector^a^						
Ultra‐Orthodox Jews	1.303	0.795–2.168	0.300	0.535	0.272–0.968	0.052
Arabs	1.882	1.291–2.771	0.001	1.714	1.226–2.392	0.002
SES						
Medium	1.228	1.027–1.468	0.024	1.320	1.081–1.610	0.006
Low	1.536	1.235–1.912	< 0.001	2.078	1.661–2.596	< 0.001
Year of study	0.987	0.948–1.028	0.530	0.987	0.945–1.032	0.574
WBC	1.086	1.072–1.100	< 0.001	1.019	1.006–1.031	0.003
CRP	1.045	1.027–1.063	< 0.001	1.016	0.998–1.034	0.087
Performance of CXR	5.093	4.107–6.353	< 0.001	2.080	1.620–2.700	< 0.001

Abbreviation: SES, socioeconomic status (reference: high SES).

^a^Reference: non‐ultra‐Orthodox Jews.

### 3.5. Antibiotic Treatment

To analyze the antibiotic treatment administered to the children with CAP, we categorized the treatments into two groups: oral antibiotic treatments and IV antibiotic treatments, which are presented in Tables 5 and 6, respectively, with additional subgroup analyses by treatment site, sex, and age category.

**Table 6:** Oral antibiotic treatment of young children with community‐acquired pneumonia by treatment site, sex, and age.

		Any oral antibiotics	Amoxicillin	Amoxicillin–clavulanate	Azithromycin	Cefalexin	Cefuroxime axetil	
*Community pediatrician (* *N* = 55,977 *)*								
Total no.		55,136 (98.5%)	33,611 (61.0%)	2823 (5.1%)	18,142 (32.9%)	208 (0.4%)	352 (0.6%)	*p* value
Sex	Female	25,723	15,804 (61.4%)	1317 (5.1%)	8331 (32.4%)	109 (0.4%)	162 (0.6%)	0.073
	Male	29,413	17,807 (60.5%)	1506 (5.1%)	9811 (33.4%)	99 (0.3%)	190 (0.6%)	
Age group	1–3 years	45,640	28,702 (62.9%)	2320 (5.1%)	14,140 (31.0%)	178 (0.4%)	300 (0.7%)	< 0.001
	4–6 years	9496	4909 (51.7%)	503 (5.3%)	4002 (42.1%)	30 (0.3%)	52 (0.5%)	
*Pediatric emergency department (* *N* = 3685 *)*								
Total no.		2432 (66.0%)	1821 (74.9%)	114 (4.7%)	446 (18.3%)	26 (1.1%)	25 (1.0%)	*p* value
Sex	Female	1093	815 (74.6%)	46 (4.2%)	204 (18.7%)	17 (1.6%)	11 (1.0%)	0.239
	Male	1339	1006 (75.1%)	68 (5.1%)	242 (18.1%)	9 (0.7%)	14 (1.0%)	
Age group	1–3 years	2051	1601 (78.1%)	98 (4.8%)	311 (15.2%)	21 (1.0%)	20 (1.0%)	< 0.001
	4–6 years	381	220 (57.7%)	16 (4.2%)	135 (35.4%)	5 (1.3%)	5 (1.3%)	

*Note:*All values are presented as *n* (percentage).

Of the children with CAP treated in the community, 55,136 out of 55,977 (98.5%) received oral antibiotics (Table 6). Amoxicillin and azithromycin were the most common antibiotics prescribed in this group (61.0% and 32.9%, respectively). Sex had no significant association with the oral antibiotic agent used; on the other hand, age had a significant effect, as higher rates of children aged 4–6 years received azithromycin than children aged 1–3 years (42.1% and 31.0%, respectively, *p* < 0.001). Of the children treated in the PEDs, 66.0% (2432 of 3685) were treated with oral antibiotics. Amoxicillin and azithromycin were the leading antibiotics prescribed also in this group (74.9% and 18.3%, respectively). Age had a significant effect on the choice of the oral antibiotics: Azithromycin was prescribed for 35.4% of children aged 4–6 years compared to 15.2% for children aged 1–3 years (*p* < 0.001). Other oral antibiotic agents were used infrequently; additional details are given in Table 6.

IV antibiotics were prescribed for 1.5% (841 out of 55,977) of children treated in the community compared to 34.0% (1253 out of 3685) of children treated in PEDs (*p* < 0.001), as described in detail in Table 7. In the community, the IV antibiotic used in nearly all children (94.8%) was ceftriaxone. In children attending PEDs, ceftriaxone was also the most common antimicrobial agent used, given to 72.2% of the children, followed by cefuroxime (19.6%), and only 6.8% received IV penicillin (Table 7).

**Table 7:** Intravenous antibiotic treatment of young children with community‐acquired pneumonia by treatment, site, sex, and age.

Antibiotics									
		Any IV antibiotics	Penicillin	Cefazolin	Ceftazidime	Ceftriaxone	Cefuroxime	Piperacillin/tazobactam	*p*value
*Community pediatrician (* *N* = 55,977 *)*									
Total no.		841 (1.5%)	11 (1.3%)	2 (0.2%)	10 (1.2%)	797 (94.8%)	21 (2.5%)		
Sex	Female	377	3 (0.8%)	1 (0.3%)	4 (1.1%)	360 (95.5%)	9 (2.4%)		0.816
	Male	464	8 (1.7%)	1 (0.2%)	6 (1.3%)	437 (94.2%)	12 (2.6%)		
Age group	1–3 years	726	8 (1.1%)	2 (0.3%)	6 (0.8%)	691 (95.2%)	19 (2.6%)		0.081
	4–6 years	115	3 (2.6%)	0 (0.0%)	4 (3.5%)	106 (92.2%)	2 (1.7%)		

*Pediatric emergency department (* *N* = 3685 *)*									
Total no.		1253 (34.0%)	85 (6.8%)	6 (0.5%)	3 (0.2%)	905 (72.2%)	246 (19.6%)	8 (0.6%)	
Sex	Female	565	40 (7.1%)	0 (0.0%)	2 (0.4%)	410 (72.6%)	109 (19.3%)	4 (0.7%)	0.328
	Male	688	45 (6.5%)	6 (0.9%)	1 (0.1%)	495 (71.9%)	137 (19.9%)	4 (0.6%)	
Age group	1–3 years	1118	66 (5.9%)	4 (0.4%)	2 (0.2%)	811 (72.5%)	229 (20.5%)	6 (0.5%)	< 0.001
	4–6 years	135	19 (14.1%)	2 (1.5%)	1 (0.7%)	94 (69.6%)	17 (12.6%)	2 (1.5%)	

*Note:*Values are presented as *N* (percentage).

## 4. Discussion

The present study provides several noteworthy findings. First, using a large population‐based cohort of young children with CAP, we determined the real‐world rate of children attending PEDs (6.2%) and used a multivariate model to identify sociodemographic and laboratory variables independently and significantly associated with PED attendance compared with evaluation by the community pediatrician. Second, we elucidated the performance of blood tests and chest radiographs in the PEDs compared to the community pediatric clinic. Third, the rate of hospitalization among children with CAP was determined by identifying real‐world variables that were independently associated with hospitalization. Fourth, we showed that children with CAP attending PEDs were indeed more ill than those managed in the community, as reflected by the markedly higher hospitalization rate (33.6% vs. 1.4%). Finally, the antibiotic regimens used to treat children in the community clinics and in the PEDs, both by oral and iv routes, were detailed.

To the best of our knowledge, this is the first comprehensive study of children with CAP who were evaluated in the PEDs, characterizing the variables associated with PED attendance and the treatment decisions. Member of a minority sector, low SES, elevated white blood cell count, and high CRP levels were significantly (*p* < 0.001) associated with PED attendance by children with CAP. These findings should be considered by the community pediatrician who evaluates young children with CAP, together with the clinical details, to optimize the management of these children, some of whom may have a severe course [4, 5, 17–20]. Moreover, identifying the associated variables and quantifying that 6.2% of children with CAP attend PEDs and 3.4% are hospitalized can aid in planning healthcare for these children.

Our findings are generally consistent with previous reports on the severity of pediatric CAP [17, 18, 21] and augment them. The higher rates of attending the PED and hospitalization of children with CAP of low SES and who belong to minority sectors are in concert with the severity of CAP in low‐ and middle‐income countries [22]. Likewise, our finding that elevated white blood cell count and serum CRP are significantly associated with attending PEDs and hospitalization corroborates previous studies that have identified these markers as indicators of severe pediatric CAP [18, 21, 23]. In particular, Williams et al. investigated the effectiveness of WBC counts and CRP levels in predicting the length of hospital stay and fever duration among 153 children with CAP aged 2 months to < 18 years [23]. They demonstrated that while WBC counts did not predict length of stay or fever duration, they may help in identifying complications in severe pneumonia, whereas CRP levels correlated significantly with hospitalization rate [23].

According to the guidelines of PIDS, the performance of white blood cell count should be reserved for severe cases of pediatric CAP [10]. The Israeli Pediatric Association Clinical Guidelines for Diagnosis and Treatment of CAP in children highlight that leukocytosis of >15 × 10^3^/*μ*L and elevated CRP levels may indicate a severe disease, although this level of leucocyte count may also occur in viral infections [12].

Our finding of the high 33.6% hospitalization rate of the children with CAP attending the PEDs is consistent with previous data. For example, a US nationwide study across 35 children′s hospitals reported hospitalization rates ranging from 19% to 69% for pediatric pneumonia diagnosed in emergency departments [17]. These findings suggest that CAP cases diagnosed in the PED likely represent a more severe clinical disease.

Regarding the performance of chest radiographs in children with CAP, we demonstrate a low rate of performance in children visiting their community pediatrician but a 91.3% performance in children with CAP attending the PEDs. These findings align with the PIDS guidelines, which discourage routine chest radiographs in children well enough to be treated in the community [10]. However, the PIDS guidelines recommend chest radiographs for all hospitalized cases without specific guidance for PED‐only evaluations [10]. Israeli guidelines similarly base CAP diagnosis on clinical assessment, with imaging reserved for suspected complications; these guidelines do not differentiate between ED‐diagnosed and community‐diagnosed cases [12]. In accordance with the guidelines, a national retrospective study assessing the use of chest x‐rays across 30 EDs in the United States showed a decrease in the use of chest x‐rays for children with pneumonia and respiratory illnesses [24]. This reduction in imaging was accompanied by a parallel decline in pneumonia diagnoses over the same timeframe [24]. In a study conducted in Finland examining the impact of Finnish guidelines on lower respiratory tract infections in children, the authors observed a notable decrease in the proportion of patients with CAP undergoing chest radiographs, dropping from 84.6% to 71.3% [25].

Antibiotic prescribing patterns also warrant attention. All children with CAP included in the present study received antibiotics. Amoxicillin was the most commonly prescribed oral antibiotic agent, followed by azithromycin. Ceftriaxone was the predominant iv antibiotic used due to its broad coverage of the usual pathogens causing pediatric CAP and also convenient once‐daily dosing. According to the PIDS guidelines, antimicrobial therapy is not routinely indicated for preschool‐aged children with CAP in the community setting, as viral pathogens are the primary cause for the majority of cases. These guidelines specify amoxicillin as the first‐line treatment for previously healthy, properly immunized infants and preschool‐aged children with mild to moderate CAP who were diagnosed in the community [10]. The Israeli guidelines also recommend amoxicillin as the preferred antibiotic treatment for suspected bacterial CAP [12]. Research on antimicrobial treatments for CAP shows that oral amoxicillin is globally accepted as the first‐line therapy [6, 10, 11].

Our results align with international prescribing patterns, though with some variability. An Italian PED study found antibiotic use in 97.2% of cases of pediatric CAP [25], while French data reported prescription rates of 83%–86% [26]. In contrast, a Dutch study reported a lower rate, with 60% of children with CAP receiving amoxicillin [27]. These differences likely reflect physician training, local practice norms, and parental expectations for antibiotic therapy [28].

The relatively frequent use of oral cephalosporins and amoxicillin–clavulanate for children with CAP treated in the community highlights the need for antibiotic stewardship programs. Indeed, Cohen et al. [28] evaluated an antibiotic stewardship program′s effectiveness on antibiotic prescriptions for children aged 3 months to 18 years with CAP. The intervention included a 1‐day seminar for primary care pediatricians on the diagnosis and treatment of pediatric CAP according to Israeli guidelines. They describe a 58% decrease in azithromycin prescribing and an increased use of amoxicillin in these younger children [28].

We also observed the lowest rate of CAP diagnoses during 2020, coinciding with the COVID‐19 pandemic. Such a decline has been reported before and attributed to decreased social interactions during the pandemic and to direct effects of SARS‐CoV‐2 on the circulation of respiratory pathogens [29].

### 4.1. Strengths and Limitations

The strengths of the present study include its population‐based design, relatively large sample size, real‐world setting, and practical relevance. To our knowledge, this is the first study that evaluated comprehensively children with CAP that were examined in the PEDs, including their demographics, diagnostic testing, antibiotic treatment patterns, and outcomes, and defined variables associated with attending the PEDs and hospitalization. The key limitation of the study is its medical records–based retrospective design that lacked clinical data, such as the findings on physical examination and the clinical severity data, which obviously inform decision‐making, including PED attendance and hospitalization. Potential diagnostic misclassification and additional confounding may play a role in the retrospective study. Also, as the organization of pediatric healthcare differs considerably by location, it is suggested to confirm the results in additional locations, preferably by prospective studies.

## 5. Conclusion

The present large population‐based real‐world study of young children with CAP determined the rates of PED attendance and hospitalization and identified sociodemographic and laboratory variables significantly associated with these outcomes. Community pediatricians should be aware of these variables and integrate them with clinical assessment to optimize management of pediatric CAP, given the potential for complications and mortality. In addition, recognizing that 6.2% of children with CAP attend PEDs and 3.4% require hospitalization, with the identification of the associations with these outcomes, has important implications for pediatric healthcare planning by policymakers.

**Nomenclature**
ANC: absolute neutrophil count
CAP: community‐acquired pneumonia
CBC: complete blood count
CRP: C‐reactive protein
HMO: health maintenance organization
PED: pediatric emergency department
PIDS: Pediatric Infectious Diseases Society
SES: socioeconomic status
WBC: white blood cell count

## Author Contributions

All authors conceptualized and designed the study and drafted the initial manuscript. Bernice Oberman performed the statistical analysis. All authors wrote sections of the first draft of the manuscript and critically reviewed and revised the manuscript for important intellectual content. Shay Nemet and Yael Reichenberg contributed equally to the work.

## Funding

No funding was received for this manuscript.

## Ethics Statement

The study protocol was approved by the Institutional Review Board of Clalit Health Services, which waived signed consent, as the study was not a clinical experiment and was based on an existing database.

## Conflicts of Interest

The authors declare no conflicts of interest.

## Data Availability

The data that support the findings of this study are available from the corresponding author upon reasonable request.
